# Immunoassays: Analytical and Clinical Performance, Challenges, and Perspectives of SERS Detection in Comparison with Fluorescent Spectroscopic Detection

**DOI:** 10.3390/ijms25042080

**Published:** 2024-02-08

**Authors:** Xeniya Terzapulo, Aiym Kassenova, Rostislav Bukasov

**Affiliations:** Department of Chemistry, Nazarbayev University, Kabanbay Batyr Ave. 53, Astana 010000, Kazakhstan

**Keywords:** SERS, fluorescence, immunoassays, LOD, sensitivity, accuracy, non-specific binding, biomarker, cancer marker, ELISA

## Abstract

Immunoassays (IAs) with fluorescence-based detection are already well-established commercialized biosensing methods, such as enzyme-linked immunosorbent assay (ELISA) and lateral flow immunoassay (LFIA). Immunoassays with surface-enhanced Raman spectroscopy (SERS) detection have received significant attention from the research community for at least two decades, but so far they still lack a wide clinical commercial application. This review, unlike any other review that we have seen, performs a three-dimensional performance comparison of SERS IAs vs. fluorescence IAs. First, we compared the limit of detection (LOD) as a key performance parameter for 30 fluorescence and 30 SERS-based immunoassays reported in the literature. We also compared the clinical performances of a smaller number of available reports for SERS vs. fluorescence immunoassays (FIAs). We found that the median and geometric average LODs are about 1.5–2 orders of magnitude lower for SERS-based immunoassays in comparison to fluorescence-based immunoassays. For instance, the median LOD for SERS IA is 4.3 × 10^−13^ M, whereas for FIA, it is 1.5 × 10^−11^ M. However, there is no significant difference in average relative standard deviation (RSD)—both are about 5–6%. The analysis of sensitivity, selectivity, and accuracy reported for a limited number of the published clinical studies with SERS IA and FIA demonstrates an advantage of SERS IA over FIA, at least in terms of the median value for all three of those parameters. We discussed common and specific challenges to the performances of both SERS IA and FIA, while proposing some solutions to mitigate those challenges for both techniques. These challenges include non-specific protein binding, non-specific interactions in the immunoassays, sometimes insufficient reproducibility, relatively long assay times, photobleaching, etc. Overall, this review may be useful for a large number of researchers who would like to use immunoassays, but particularly for those who would like to make improvements and move forward in both SERS-based IAs and fluorescence-based IAs.

## 1. Introduction

Immunoassays are analytical methods that contribute to the determination of the contents of specific compounds in samples by exploiting antigen–antibody interactions [1]. Antigens are compounds that induce the synthesis of antibodies and are specifically recognized by glycoproteins called antibodies [2]. Immunoassays have extremely high specificity. They are widely used in areas such as toxicology, endocrinology, cardiology, and infectious diseases [1]. One of the first assays based on the binding of antigens to antibodies, known as radioimmunoassay, was developed by scientists Yalow and Berson in 1960. A radioimmunoassay was used wherein the radioactivity of beef insulin −I^131^ was measured to calculate the concentration of human insulin from its standard addition to the mixture [3]. There are many types of immunoassays available in this field today. They include radioimmunoassays (RIAs), enzyme immunoassays (EIAs), fluorescence immunoassays (FIAs), chemiluminescence immunoassays (CLIAs), nonlabeled immunoassays, electrochemical immunoassays, liposome immunoassays, chromatographic immunoassays, electrophoretic immunoassays, SERS-based immunoassays, and miscellaneous immunoassays [4]. We provide a detailed description of fluorescence-based immunoassays and SERS-based immunoassays in the following sections.

### 1.1. Fluorescence-Based Immunoassays Detection

Fluorescence spectroscopy is increasingly used to analyze both the physical and chemical behaviors of macromolecules, allowing for the high sensitivity and comparatively low quantitative detection that makes it an appropriate tool for trace analysis [5]. In fluorescence spectroscopy, an electron in a fluorophore molecule gets excited to a higher electronic state after the absorption of a photon. Then, the electron relaxes from an excited electronic state to one of the multiple vibrational levels of the ground electronic state with the emission of a photon, which makes up fluorescent light [6]. The fluorescence relies on the ability of the compound/fluorophore to absorb light energy at a particular wavelength and reemit light at a longer wavelength, while each compound has two characteristic spectra: an excitation spectrum and an emission spectrum [6]. These spectra form the unique fluorescence signature for each compound that increases the specificity of the analytical performance of the fluorescence-based detection techniques [6]. Photomultipliers (PMTs) are often used as detectors in the fluorescence method, allowing them to achieve high sensitivity due to their ability to detect very low light intensity [7].

Enzyme immunoassays are often used in conjunction with enzyme-linked immunosorbent assays (ELISA) in biomedical research due to their ability to qualitatively and quantitatively determine the content of macromolecules in the studied samples. This feature helps in early disease detection due to high sensitivity. There are several types of ELISA, the most common of which are indirect and sandwich ELISAs. Both types allow for the detection of analytes at low concentrations [8]. Engvall et al. suggested coupling enzymes to the antibodies for the histochemical detection of the antigens in the tissues [9]. In the same year, Van Weemen et al. also underlined the high sensitivity of the enzyme immunoassays by coupling the antigen human chorionic gonadotrophin (HCG) to the enzyme horse radish peroxidase (HRP) [10].

The enzymatic immunoassay methods are classified into two major divisions—homogeneous and heterogeneous [11]. Homogeneous immunoassays do not require the separation stage for antibody-bound complexes, while heterogenous immunoassays involve the washing stage for the separation of the complexes [11]. The enzyme-linked immunosorbent assay is the heterogeneous enzymatic immunoassay that has the antigen–antibody complex bound to the walls of the experimental tubes to separate it from the washing procedure [11]. For different types of analytes, the ELISA methods are also divided. For example, large molecules with different epitopes can be detected using the sandwich ELISA, whereby the antigen is positioned between two antibodies (capture and detection). This method requires several wash steps that remove the unreacted antigen and antibody [12]. The highly weighted antigens are detected using either direct or indirect ELISA, whereby the analyte is deposited directly on the surface of the plate, and antibodies are added on top [11]. In the direct method, only one set of labeled antibodies is used. In the indirect method, unlabeled primary and labeled secondary antibodies are used [11]. As for the detection of small molecules, competitive ELISA is applied, which is based on the competitive reaction happening between the enzyme-labeled antigen and the analyte [12].

Posthuma-Trumpie et al. noted the diagnostic relevance of lateral flow assays, as it they are used for the detection of infectious diseases, contamination with specific pathogens, and food contamination [13]. The basic principle of this analytical technique involves using a fluid sample to activate the dry reagents on pre-made strips in lateral flow immunoassay (LFIA) [13]. The main components of the LFIA are the chromatographic system separating the mixture components based on their movement through the membrane of the reaction, and the immunochemical reaction happening between the antibody and antigen [14]. The movement of the liquid sample is accomplished across the membrane via capillary force [14]. It is reported that Plotz and Singer developed the operating basis of the LFIA in 1956, and based it on the latex agglutination assay, at the same time as the development of plate-based immunoassays [15].

### 1.2. SERS-Based Immunoassays Detection

Surface-enhanced Raman spectroscopy (SERS) is a powerful technique for the highly sensitive detection of low-concentration molecules [16]. SERS endows the ability to identify molecules by obtaining their molecular formulas. This method can be applied in various fields, such as medicine, the food industry, and material science, due to its high sensitivity and selectivity [16]. It can be also used in the detection of biomarkers of such diseases as cancer, Alzheimer’s, and Parkinson’s [16].

There can be direct and indirect SERS detection configurations [17]. Direct SERS does not apply a sensing molecule that would bind to the analyte molecule (e.g., antibody, aptamer) and the analyte is bound/absorbed to the substrate directly [18,19]. In comparison to direct SERS, indirect SERS, including immunoassays with SERS detection, typically allows a better limit of detection (LOD), at least in bioanalytical applications, because there is better binding of the analyte to the substrate with the help of a capture antibody or aptamer. LOD is typically defined as the lowest reliably detected concentration of an analyte. The advantages of indirect SERS in comparison with direct SERS may also include less complicated Raman peaks and multiplex ability.

The first mention of SERS in research was made by Fleischmann et al. in 1973 when studying pyridine and its adsorption on the surface of silver, while SERS-based IAs were first reported in 1999 using the “sandwich” scheme [19,20]. Van Duyne explained the electromagnetic mechanism of SERS enhancement in 1976, when he identified the difficulties of the detection of reaction intermediates and other molecules using electrochemical methods of detection [19]. The popularity of SERS-based immunoassays in research has grown significantly throughout the last few decades due to the advantages of this method. These advantages include high sensitivity, relative resistance to photobleaching and absence of quenching, stability of signals, as well as high throughput created by spectroscopic coding [20]. Sandwich SERS immunoassays, where the antigen (analyte) is sandwiched between the capture antibody on the substrate and the detection antibody on the nanotag (AuNP modified with Raman reporter and detection antibody), have been used to detect a variety of biomarkers by more than several research groups [20]. These advantages distinguish SERS-based immunoassays from other techniques. It was determined that SERS-based aptamer assays are a little bit less sensitive than SERS-based immunoassays. Immunoassays are a more well-established method for the detection of biomolecules than aptamer-based assays, and on average, they are also more sensitive. The mean values of LODs calculated in 88 reports of immunoassays and 51 reports of aptamer-based assays were 0.5 pM and 1.7 pM, according to Iliyas et al. [21]. The detection of femtomolar concentration of prostate-specific antigen (PSA), a biomarker for prostate cancer, performed by Porter’s group about two decades ago, exemplified the very high sensitivity of this method [22]. In addition, Porter’s group have demonstrated the versatility of immunoassays with SERS detection for a range of bio-analytes, for instance, the detection of biomarkers for cancer screening and tuberculosis, bacteria, toxins, and viruses [23,24,25,26,27,28]. There is good potential for extensive multiplexing via the SERS methods due to the fact that the spectral overlap between various Raman labels is minimized by the narrow widths of Raman spectral features, which are 10–100 times narrower compared to the spectral features of fluorescence [29].

Since reproducibility is considered to be one of the key challenges related to SERS biosensing, Crawford et al. studied how to improve it for sandwich immunoassays with SERS detection and suggested using a larger laser spot as a solution [30]. Owens et al. recently reported surface-enhanced resonance Raman scattering (SERRS) in immunoassays for which Raman Reporter Molecule (RRM) coating was located not on Extrinsic Raman Label (ERL) nanotags, but rather on the capture substrate; they reported a 10–40-fold improvement in LOD and analytical sensitivity in the detection of TB biomarker mannose-capped lipoarabinomannan (ManLAM) [31]. In an attempt to decrease SERS-based assays’ costs, non-noble metal substrates were tested in sandwich SERS immunoassays. For instance, Bukasov et al. proposed aluminum foil as a very economical and stable substrate for use in the detection of tuberculosis biomarker MPT64. The performance of the assay on Al foil was compared with its performance on gold film. The assay on Al foil had better selectivity/lower non-specific binding and sometimes lower LODs than those obtained for the assay on gold film [32].

In addition to fluorescent and SERS-based methods, methods such as vibrational spectroscopic techniques can also be used. Fourier Transform Infrared (FTIR) spectroscopy is one such technique. FTIR is a conservative technique, by which the functional groups, bonds, and molecular conformations of the studied compounds can be identified [33]. According to Aitekenov et al., SERS is about three orders of magnitude more sensitive than FTIR, and there are only a few publications on immunoassays with FTIR detection [34]. Therefore, in this review, we focus on comparing the performances of SERS-based immunoassays to those of fluorescence-based immunoassays. In the second part of the review, we discuss common and specific challenges of both techniques and propose solutions for these challenges.

## 2. Analytical Reports on Immunoassays with the SERS and the Fluorescence Based Detection

### 2.1. Analytical Papers on the Fluorescent Immunoassays

**Figure 1:**
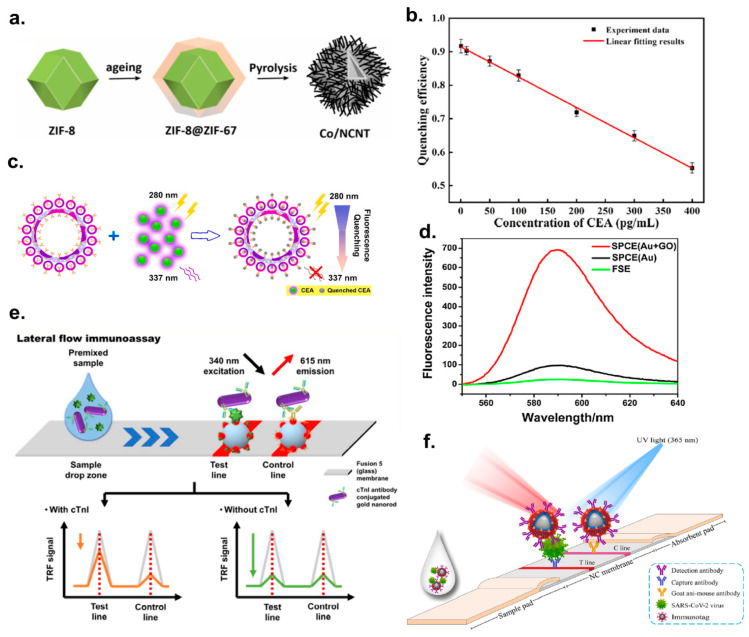
Analytical papers based on fluorescent immunoassay detection. (**a**) The scheme of formation of the Co nanoparticle and N-doped carbon nanotubes (Co/NCNT) by pyrolysis of ZIF-8@ZIF-67. Adopted from Chen et al. [39] Copyright © 2022 Elsevier B.V. (**b**) The graphic representation of the quenching efficiency of hollow porous gold nanoshells (HPGNPs), anti-CEA (carcinoembryonic antigen) modified and tagged with 4-mercaptobenzoic acid (4-MBA), differing with changes in the CEA concentration. Adopted from Yang et al. [68] Copyright © 2021 Elsevier B.V. (**c**) The scheme of detecting CEA using anti-CEA modified 4-MBA-tagged HPGNPs@ based on fluorescence quenching. Adopted from Yang et al. [68] Copyright © 2021 Elsevier B.V. (**d**) The graph of the fluorescence spectra of surface plasmon-coupled emission (SPCE) with graphene oxide (GO) on gold film and of free space emission (FSE) without GO. Reused from Xie et al. [42] Copyright © 2017 Elsevier B.V. (**e**) The schematic principle of LFIA using the fusion 5 membrane as a strip and the time-resolved fluorescence energy transfer (TR-FRET) technique. Adopted from Lee et al. [67] Copyright © 2020 Elsevier B.V. (**f**) A schematic illustration of a dual-functional LFIA biosensor. Reused from Han et al. [52] Copyright © 2022 Elsevier B.V All permissions to use copyrighted images were obtained.

To discuss the overall performance of fluorescence-based immunoassays, the results reported in forty recent papers (most are from 2019–2022) with LOD and other Figures of Merit (FoM) have been used to construct Table 1. Some of those papers and notable FIA approaches/examples are illustrated in Figure 1. The detection of the most common biomarkers used as analytes and the classifications of fluorescent detection are discussed below.

Figure 1 illustrates the fluorescence immunoassay methods. Most of the analyzed papers note the limited selection of analytes. For example, the oxidase-mimicking reagent, which is Co/NCNT, is formed by the pyrolysis of the ZIF-8@ZIF-67, as shown in Figure 1a [39]. The dual immunoassay was designed with colorimetry and fluorescence, using the precursor Co/NCNT with improved oxidase-mimicking activity. This is because each of the colorimetry and fluorescence immunoassays demonstrated limited efficiency and limited sensitivity for the detection of ochratoxin A. The hollow structure shown in Figure 1a assures available active sites by enhancing Co/NCNT’s catalytic activity. Figure 1b shows that the efficiency of the fluorescence quenching of 4-MBA on anti-CEA nanoparticles decreases from 0.92 to 0.54 when the concentration of carcinoembryonic antigen (CEA) increases from 0.5 pg/mL to 400 pg/mL [68]. In Figure 1c, the fluorescent probe, anti-CEA-modified 4-MBA-tagged HPGNPs@mSiO_2_-Ring, was added for the detection of CEA, accomplished with metal-induced fluorescence quenching [68]. Figure 1d demonstrates the fluorescence spectra produced when surface plasmon-coupled emission (SPCE) is assisted with graphene oxide (GO) on Au film. In their work, Xie et al. explained the use of SPCE-based methods due to their enhanced fluorescence signal, which is a result of the increased collection efficiency for sensitive protein detection applications. The adsorption of the GO on the gold film is a homogeneous process that ensures its high stability as a substrate [42]. Lee et al. highlighted the higher sensitivity of fluorescence-based LFIA for the detection of cTnI with a lanthanide dye due to its long fluorescence decay time and large stoke shift, preventing interference with autofluorescence, as shown in Figure 1e. Fluorescence-based LFIA is often preferential to conventional LFIA since the latter typically has relatively low sensitivity for the analytical detection of cardiac biomarkers. The aforementioned fluorescence-based assay engages the fluorescence energy transfer (FRET) process, which explains the increased fluorescence in the presence of cardiac troponin I, and the correspondingly decreased signal when the following analyte is absent [67]. The design of colorimetric fluorescent SiO_2_@Au/QD NPs for the effective detection of SARS-CoV-2 S1 is demonstrated in Figure 1f [52,68]. Here, the LFIA-based detections of targeting S proteins are carried out with relatively rapid responses due to their dual functionality [52]. The LFIA detection of SARS-CoV-2-S1 has lower sensitivity, and QDs were proposed for use as the fluorescent material due to their simple synthesis, low cost, and mass production capacity [52]. The biosensor outlined in Figure 1f provides more accurate monitoring for SARS-CoV-2 infection [52].

### 2.2. Analytical Papers on the SERS Immunoassays

Table 2 reviews the major FoMs of 40 analytical papers on immunoassays with SERS detection. Based on calculations from the 30 studies shown in the paper, when LOD could be recalculated as molarity, the median LOD was 4.3 × 10^−13^ M, while the geometric mean was equal to 5.4 × 10^−13^ M.

Surface-enhanced Raman scattering (SERS) spectroscopy is often used to determine the concentration of the analyte in the sample with accuracy at a single-molecular level. The analysis is often carried out using different objectives and excitation lasers. Among the 40 publications mentioned above, almost half of them used a 785 nm laser; a 633 nm laser was used less often, and the least popular was the 532 nm laser. This trend may indicate that there is a possibility that a 785 nm laser may be more suitable for the detection of proteins and other biological molecules. In the above-mentioned works, the 20× objective was likely used most frequently; a 50× objective was used a bit less frequently, and 10× and 100× lenses were also sometimes used.

One of the most sensitive sensors is the SERS-based sandwich immunoassay, reported by Yang et al. This group presented the core–shell–satellite nanostructure (Au@Ag@SiO_2_–AuNP), the LOD of which was equal to 4.4 × 10^−18^ M [92]. This biosensor was manufactured for the detection of alpha-fetoprotein (AFP), which is known as a biomarker of cancer. Another sensor, used for the detection of Human Immunoglobulin G (HIgG), was distinguished by its high sensitivity. Song et al. presented a SERS-based immunoassay, the LOD of which was equal to 6.7 × 10^−18^ M [90].

Langer et al. outlined the perspectives of SERS with microfluidic approaches [113]. Combined Lateral Flow Immunoassays (LFIAs) attracted the attention of several prominent research groups including Tang’s group, Zhang’s group, Tamer’s group, Wang’s group, Xiong’s group, Cai’s group, and Sun’s group, whose works are presented in Table 2 [77,78,83,95,98,104,111]. In general, assays used in the studies presented in Table 2 were divided into three categories: stagnant on solid substrate, LFIA, and in solution. The median and geometric mean of LOD for each type of assay are presented in Table 3.

All studies focused on these calculations are shown in Table 2. Overall, the differences in median LOD values for all three categories are found to be approximately one order of magnitude. Surprisingly, the methods with stagnant on solid substrate were found to be the most sensitive, and their median LOD was equal to 1.2 × 10^−13^ M. The median LOD values for LFIA and for assays in solution were equal to 1.2 × 10^−12^ M and 3.1 × 10^−12^ M, respectively. Together with sensitivity, the time for detection is also found to be an important factor in the comparison of the analytical papers. The best reported assay time for SERS-based LFIA was presented by Jia et al. and was equal to 15 min [95]. Deng et al. presented the fastest detection method using the stagnant on solid substrate assay, and it was equal to 15 min [112]. The inverse trend between sensitivity and assay time should be mentioned, since the fastest assay was presented by Choi et al. and was equal to 10 min using the SERS-based microdroplet platform, whereas the median LOD for assay in solution was found to be lower than for previously mentioned types of assays [91].

**Figure 2:**
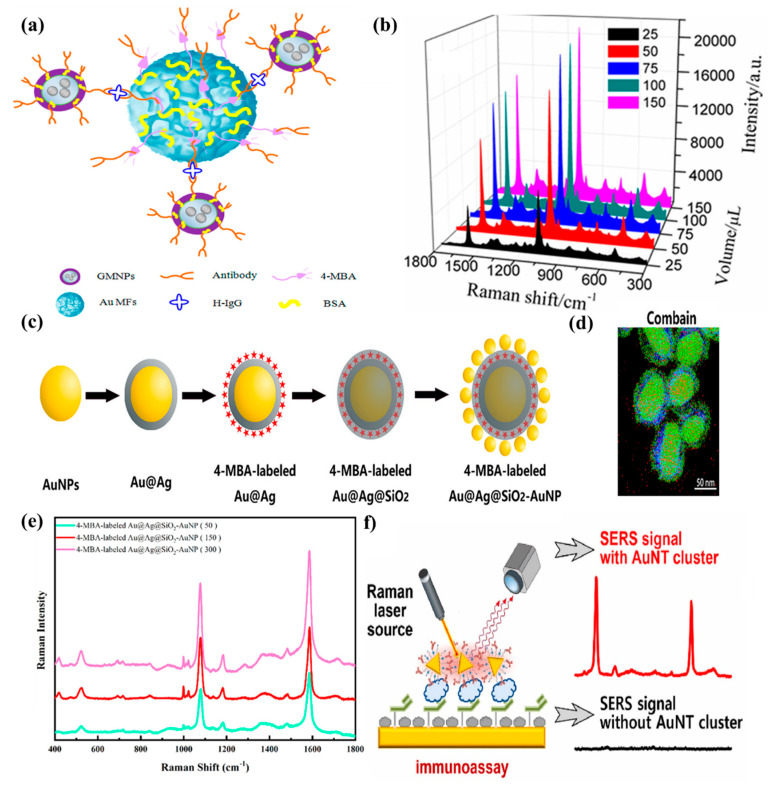
Analytical papers on SERS-based immunoassay detection of analytes. (**a**) Representation of the 4-MBA-labeled Au MFs sandwich immunoassay. Adopted from Song et al. [90] Copyright © 2014, American Chemical Society. (**b**) Characterization of 4-MBA-labeled Au MFs by mixing the Au MFs with 1 mM 4-MBA in different volumes by SERS measurements. Adopted from Song et al. [90] Copyright © 2014, American Chemical Society. (**c**) Schematic representation of the 4-MBA-labeled Au@Ag@SiO_2_–AuNP nanoassemblies’ preparation. Adopted with permission from Yang et al. [92] Copyright © 2019, American Chemical Society. (**d**) Energy-dispersive X-ray spectroscopy (EDS) mapping of Au@Ag@SiO_2_–AuNP nanoassemblies. Adopted with permission from Yang et al. [92] Copyright © 2019, American Chemical Society. (**e**) SERS spectra of 4-MBA-labeled Au@Ag@SiO_2_–AuNP nanoassemblies coated with silver with different thicknesses. Adopted with permission from Yang et al. [92] Copyright © 2019, American Chemical Society. (**f**) Scheme of the quantitative characterization of adiponectin using AuNT cluster-based SERS immunoassay. Adopted with modification from Kim et al. [88] © 2022 Elsevier B.V. All permissions to use copyrighted images were obtained.

Figure 2 illustrates analytical studies based on the detection of analytes using SERS-based immunoassays. Figure 2a demonstrates a schematic illustration of the sandwich immunoassay used to determine human IgG in samples [90]. To obtain such an assay, Song et al. mixed SERS tags with SERS immuno-GMNPs, followed by human immunoglobulin G (IgG) antigens. Next, it was necessary to leave the sample for incubation for 3 h and then rinse it three times with borate buffer solution (BBS), after separating everything with a magnet. To carry out SERS measurements, the sample had to be diluted with water. To check how the Raman spectra of golden mesoflowers (Au MFs) change depending on the increase in the volume of 4-Mercaptobenzoic acid (4-MBA), SERS measurements of five samples were carried out, in which 25, 50, 75, 100, and 150 µL of 1 mM 4-MBA were added to 1 mL of Au MFs. The results of these measurements are presented in Figure 2b [90]. According to these spectra, it can be noted that with the changes in the volume of 4-MBA, there are similar shifts of signals, but nevertheless, the intensity of the peaks is significantly different. Figure 2b illustrates that when adding 150 µL of 1 mM 4-MBA to Au MFs, the signal was more intense than in those samples in which 25 µL of 1 mM 4-MBA was added. Song et al. presented their work in which the detection of Human IgG was performed using SERS-based immunoassays, and the limit of detection for this work was equal to 6.7 × 10^−18^ M. An illustration of the process of forming core–shell–satellite nanoassemblies (Au@Ag@SiO_2_–AuNP nanoassemblies) is shown in Figure 2c [92]. To do this, gold nanoparticles with a diameter of 50 nm were covered with a layer of silver. Next, the 4-MBA was attached to the AuNP with a silver-coated layer. To stabilize the solution, 4-MBA-labeled Au@Ag particles were coated with a layer of silicon oxide SiO_2_ using the Li process. A thin layer of silicon oxide (5 nm) increases the stability of the compound and endows a great surface enhancement. Satellite AuNPs were applied to the surface of the complex, which enhance electromagnetic coupling and the SERS effect. In addition, this step helps to improve the biological detection of analytes. The energy-dispersive X-ray spectroscopy (EDS) mapping of Au@Ag@SiO_2_–AuNP nanoassemblies is presented in Figure 2d [92]. It contains an image of Au@Ag@SiO_2_–AuNP nanoassemblies used for the detection of alpha-fetoprotein (AFP). This work was found to be the most sensitive among the abovementioned analytical papers, and its LOD of AFP was equal to 4.4 × 10^−18^ M. According to the image, the main density of gold was found in the inner part; for silver, it was closer to the outer border, while in silicon it was evenly distributed throughout the sample in a relatively small concentration. To check the dependence between the thickness of this sensor’s silver coating layer and the intensity of the Raman signal, SERS measurements were carried out, the results of which are presented in Figure 2c [92]. The volumes of silver nitrate (AgNO_3_) added to the sensor were 50, 150 and 300 µL, and the thickness of the silver layer was more than 3.5 nm. According to the results of the SERS measurements, the intensity of the peaks of 4-MBA-labeled cores increased in accordance with an increase in the thickness of the silver layer. The quantitative characterization of the adiponectin scheme is presented in Figure 2f [88]. Kim et al. presented an analytical paper with a limit of detection equal to 1.0 × 10^−17^ M. Here, the analyte, adiponectin, is sandwiched between monoclonal capture antibodies on the substrate and monoclonal antibodies attached to a gold nanotriangle nanotag. With an increase in the amount of adiponectin, anisotropic gold nanotriangles (AuNTs) with Raman reporters formed clustered structures, which contributed to the enhancement of the SERS effect. Accordingly, the lower the concentration of the desired protein, the worse the intensity of the peaks that were presented as a result of the analysis.

### 2.3. Clinical Papers on the SERS- and Fluorescence-Based IA Detection

Table 4 illustrates the overall performances of SERS and FIA methods in clinical applications. The reported parameters of clinical sensitivity, specificity, and accuracy are analyzed in Table 4, from which we concluded that the general performance of SERS detection is higher than that of the FIA method.

For the comparison, pathogen detection using both SERS and FIA methods can be considered: the performance in relation to the detection of influenza is lower in terms of all three parameters when the FIA method is applied, as compared to the SERS method. Influenza A and B require rapid detection and diagnosis, as these viruses cause contagious respiratory illnesses [128].

In addition, SERS has emerged as a cancer detection tool, broadly applied in clinical diagnostics. Comparatively high sensitivity, specificity, and accuracy parameters suggest its applicability in the detection of cancer biomarkers. One of the applications of the SERS method is in the detection of exosome analytes that serve as novel pancreatic cancer biomarkers. In their work, Li et al. highlighted the issue of the early detection of pancreatic cancer without sensitive biopsy methods, and proposed “an ultrathin polydopamine-encapsulated antibody reporter Ag (shell)–Au (core) multilayer (PEARL) surface-enhanced Raman scattering (SERS) nano-tag” with an established LOD of 9 × 10^−19^ M at a quantitative Raman signal of 1072 cm^−1^ [117]. Sensitivity to differentiating metastasized tumors from metastasis-free tumors was achieved [117].

According to Table 4, Lu et al. highlighted the clinical applications of fluorescence immunoassays in the detection of the brucella antibody, which can be utilized for the detection of brucellosis, an infectious zoonotic disease, noting the limited performance of the assay for the diagnosis [124]. They developed a time-resolved LFIA with an LOD of 0.3 IU/Ml for the Brucella antibody [124]. The sensitivity, specificity, and accuracy parameters calculated for the rapid method of brucellosis detection are 98.57%, 100% and 99.63%, respectively.

**Figure 3:**
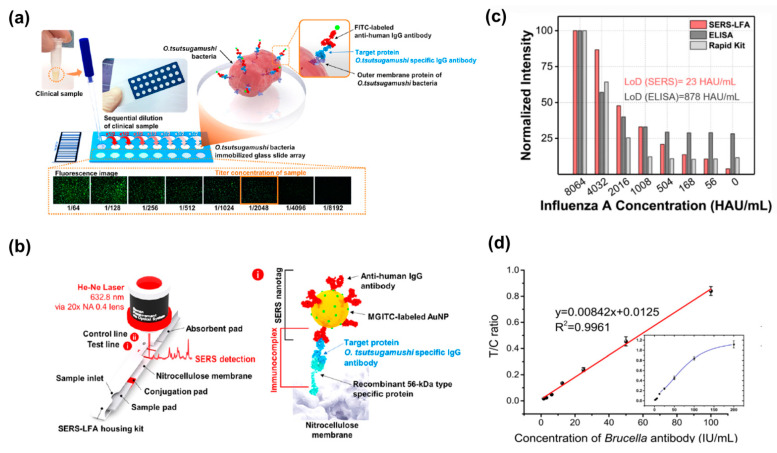
Examples of the clinical applications of SERS- and FIA-based detections. (**a**) The schematic representation of IFA for the detection of the *O. tsutsugamushi*-specific IgG antibody in the clinical sample. Adopted from Lee et al. [122] Copyright © 2019 American Chemical Society. (**b**) A schematic illustration of the operation of SERS-LFA detection for the *O. tsutsugamushi*-specific IgG antibody. Adopted from Lee et al. [122] Copyright © 2019 American Chemical Society. (**c**) A diagram of normalized intensity for various influenza A concentrations including SERS-LFA and ELISA methods. Reused form Lu et al. [121] under Creative Common License. (**d**) The graph of the curve for *Brucella* antibody detection by TF-LFIA. Adopted from Lu et al. [124] Copyright © 2021 Elsevier V.B. All permissions to use copyrighted images were obtained.

The detection of the *O. tsutsugamushi*-specific IgG antibody showed comparatively high performance in terms of the mentioned parameters in the clinical application of fluorescence immunoassays, the process of which is illustrated in Figure 3a: the *O. tsutsugamushi* bacteria were pre-immobilized on the array well surface, with the following dilution titer of the serum sample placed in each well [122]. The antibodies were then captured by the bacteria on the surface. To form a sandwich complex by antibody–antibody interaction, fluorescein isothiocyanate (FITC)-labeled antihuman IgG antibodies were added and antibody titer values were determined using the fluorescence microscope images, the clinical serum of which was serially diluted. This serial dilution weakens the intensity of the fluorescence. The figure demonstrates the usage of fluorescence microscopic images to visualize the titer values, as the end point is taken whenever it is impossible to observe the titer value visually. Thereby, the challenges associated with the fluorescence microscope diagnosis are addressed in SERS-LFA detection, the working scheme of which was presented by Lee et al. For the sensitive diagnosis of scrub typhus, the specificity of the target antibody was increased by synthesizing an *O. tsutsugamushi* recombinant protein instead of using bacteria. As shown in Figure 3b, this 56 kDa protein was synthesized using strains such as Karp, Kato, and a Gilliam, immobilizing them on the test line of the LFA strip [122]. Another example of achieving better performance in detection is represented in Figure 3c. A 40-fold improvement in sensitivity was observed for SERS-LFA in comparison with the ELISA method, the LODs of which are 23 HAU/mL and 880 HAU/mL, respectively [121]. Figure 3d also outlines the performance of TF-LFIA for the detection of the Brucella antibody, with a linear range of 1.6–100 IU/mL and an LLOQ of 1.6 IU/mL [124].

### 2.4. SERS vs. Fluorescence Immunoassays for Detection of Biomarkers

The analytes that were used in studies using both SERS-based IA detection and fluorescence IA detection are presented in Table 5. To perform these calculations, 20 stages were undertaken, in which the following compounds were used as the analytes: Aflatoxin B1, (AFB1), Ochratoxin A (OTA), Alpha-fetoprotein (AFP), and Cardiac Troponin I (cTnI). To compare the sensitivity of these methods, the medians and geometric means of the limits of detection (LODs) presented in these papers were calculated.

These analytes are quite common in connection with their applications. Aflatoxin B1 (AFB1) is a mycotoxin, which is a fungal metabolite capable of contaminating food supply in some areas of the world [130]. Derivatives of aflatoxin can cause DNA mutations, while AFB1 itself is a carcinogen and is associated with the cause of hepatocellular carcinoma (HCC). The second common analyte is Ochratoxin A (OTA), which is also recognized as a mycotoxin. This toxin is very common, as it is produced as a result of the growth of fungi on cereals, and can also be found in pork meat, sausages and other products that contain blood [131]. OTA is a possible carcinogen for humans and the strongest carcinogenic compound for mice and rats. In addition to these mycotoxins, Alpha-fetoprotein (AFP) is a common analyte. AFP is the most common biomarker of hepatocellular carcinoma (HCC) in blood samples, and has been considered as such since its discovery and assessment about 60 years ago [132]. The fourth of the analytes presented in Table 5 is the cardiac marker Cardiac troponin I (cTnI). It is extremely important to monitor the level of this marker in the blood, as it has a special sensitivity and can help in the measurement and identification of cardiac muscle damage. An increased level of Cardiac troponin I (cTnI) can help in the diagnosis of myocardial infarctions [133]. In connection with the above information, the importance of the early and sensitive detection of these analytes can be noted. The median LOD and geometric mean for SERS-based IA detection methods for all the mentioned biomarkers are 1.8 × 10^−12^ M and 1.3 × 10^−12^ M, respectively, while the same parameters for fluorescent IA-based methods are 1.3 × 10^−11^ M and 7.5 × 10^−12^ M. In most cases, the difference between the methods was no more than one to two orders of magnitude, but in the case of Alpha-fetoprotein (AFP) detection, the difference was significant. The median LOD for SERS-based IA methods for detecting this cancer biomarker was 6.0 × 10^−16^ M, while for fluorescent spectroscopic IA detection, the median LOD was 3.2 × 10^−12^ M. If in the detection of AFB1, AFP and cTnI, methods using SERS was more sensitive and the median LOD was smaller, then in the case of OTA, fluorescent spectroscopic detection was more sensitive. The median LOD for the detection of OTA by SERS-based methods was 3.5 × 10^−12^ M, while for fluorescent spectroscopic detection, it was 3.6 × 10^−13^ M. According to the results, it can be concluded that fluorescent spectroscopic detection methods are 10 times more sensitive than SERS-based methods when detecting OTA. Despite this, based on the results of our calculations, it can be concluded that the methods that use SERS-based immunoassays are more sensitive in most cases. This difference may indicate that SERS-based detection may be a more productive method for use in the process of detecting biomarkers, making it possible to obtain more sensitive sensors with the lowest detection limit.

### 2.5. RSD Discussion

Table 6 shows the average values of relative standard deviation (%RSD) for immunoassays with SERS and fluorescence as the detection methods.

Relative standard deviation (RSD or %RSD) is the absolute value of the coefficient of variation, or the ratio of absolute standard deviation to the mean value. According to Horvitz Trumpet, when the LOD/concentration of detected analyte decreases, we can expect some increase in the CV/RSD of the method [134]. According to the data presented in Table 6, the median LOD calculated in publications with SERS-based IAs, where RSD is reported, is 1.5 × 10^−12^ M, but the LOD calculated for fluorescent immunoassays with reported RSD is 2.2 × 10^−11^ M. Both methods show similar median relative standard deviations (5.4–6.2%), with a slight advantage (0.8% lower) for FIA. However, the median LOD for the reported SERS-based immunoassays is nearly one order of magnitude lower than the median LOD calculated for the fluorescence-based immunoassays, which also have reported RSDs. The analysis of the reported RSDs, summarized above, does not suggest that SERS methods generally have relatively low reproducibility in comparison with fluorescence-based methods.

## 3. Challenges and Solutions for SERS and Fluorescent Detection

### 3.1. Common Challenges and Solutions for Both SERS and Fluorescence-Based Detection

Sandwich immunoassays with SERS and fluorescent detection encounter some common challenges, such as non-specific binding, environmental sensitivity, protein adsorption, etc. Non-specific binding (NSB) in immunoassays is often found in both SERS and fluorescent methods used for the detection of biological samples or complex matrices. It is defined as any binding that differs from the specific binding of the capture antibody and capture antigen, due to various interactions between capture or secondary antibodies and the gold surface of the nanotag, and can be associated with the presence of heterophile antibodies. In SERS, this is manifested by the adsorption of unwanted parts of the samples onto the substrate, which can lead to undesirable signals on the Raman spectrum [135]. The manifestation of non-specific binding in the spectrum can lead to signal interference between non-specific peaks and analyte peaks, which in turn significantly complicates the analysis of specific and non-specific signals. In fluorescent detection, non-specific binding is manifested through background signals that may appear due to autofluorescence or as a result of the non-specific binding of fluorescent labels to the surface or other components of the sample [136]. This may lead to the appearance of background noise, as well as provoking the appearance of false-positive and/or false-negative analysis results [135].

**Figure 4:**
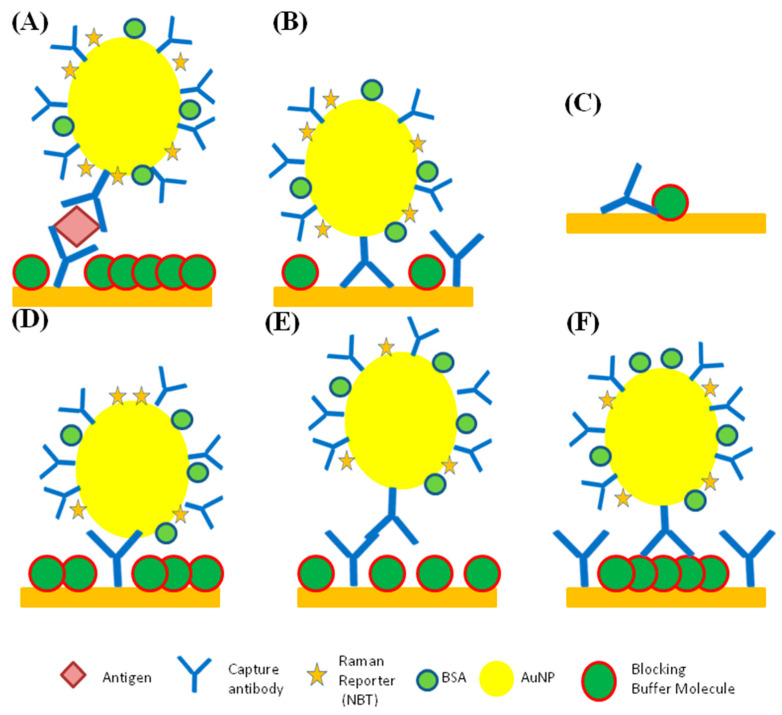
Schematic representation of specific and non-specific binding. (**A**) specific, (**B**,**D**–**F**) various kinds of non-specific (NS) nanotag binding that can produce SERS signals, (**C**) capture antibody NS binding to the substrate and blocking buffer (not to scale).

Figure 4 illustrates various options for the specific and non-specific binding (NSB) of the capture antibodies that can produce a SERS signal. Part (A) of Figure 4 illustrates the correct variant of specific binding, form which a reproducible result can be obtained. Even with the total absence of analyte in SERS, non-specific binding creates signals in the absence of antigen, and there at least four different possibilities for the occurrence of non-specific binding. For instance, as shown in Figure 4B, in the sandwich SERS immunoassay, it can happen due to the capture antibody from the nanotag binding to the surface of the substrate. We expect a decrease in non-specific absorption due to a decrease in Van der Waals interactions from gold–gold to gold–aluminum interactions, and due to the weaker S–Si or S–Al bonding than S–Au bonding of S-containing aminoacids to the substrate [137]. For instance, it was reported that the adsorption energy of organic sulfur is 0.7 eV for cysteine on gold, as compared to 0.5 eV for thiophene on Al [138,139].

The application of Al, which is also covered with a several-nanometer-thick passivating layer of Al_2_O_3_, and using Si instead of gold as an immunoassay substrate, would decrease NSB in at least two cases, as shown in Figure 4B,C. In the case of Figure 4B, there would be lower potential for the binding of the capture antibody on the nanotag to the uncovered regions on the substrate. The non-specific interaction between the capture antibody from the nanotag and bare spots on the substrate is likely to be stronger when the substrate is gold rather than aluminum or silicon. A major contribution to that kind of NSB should arise from S–Au bonding, which should be stronger than S–Si or S–Al interactions. This hypothesis is supported by the SEM and AFM characterization of sandwich SERS immunoassays on gold, Al and Si, where the nanotag particle densities for blank (no-antigen) samples were substantially higher on gold substrate than on Al and Si substrates. For instance, 0.402 and 0.226 nanotags/µm^2^ were calculated for blanks on gold and Al foil, respectively [32,137].

The case when the capture antibody loses its activity due to its non-specific binding to the substrate and to the blocking buffer protein is represented in Figure 4C. This case would also be more likely when the substrate is gold rather than aluminum or silicon. Other variations of NSB are shown in Figure 4D,E, where the capture antibody from the substrate binds to the bare surface of the gold nanoparticle (nanotag), and where the capture antibody from the nanotag binds to another antibody from the substrate, in the absence of antigen. Moreover, the binding of the capture antibody on the nanotag to the blocking buffer protein molecule on the substrate can occur, as shown in Figure 4F.

To eradicate this source of error, it is necessary to introduce into the process additional washing, analysis with a control sample, and the use of selective labels or blocking agents [32,137]. It is also expected that by switching to aluminum and silica, it would be possible to reduce such cases of non-specific binding. The results reported by Bukasov et al. illustrate that assays on aluminum and silica substrates produce a comparatively better limit of detection (LOD) than simultaneous assays of human IgG on gold [32,137]. The non-specific signal measured was about 1.8 times lower on aluminum foil and about one order of magnitude lower for silicon than for gold [32]. Human IgG assays on Al foil and Si showed better selectivity and lower non-specific responses to rat or rabbit IgG in comparison to assays using gold film. This may be explained by the roughly two times lower relative standard deviations for the signal on silicon than for the gold film substrate. Despite the lower signal and a lower slope of the calibration plot in the assay on silicon, the reproducibility of this substrate is better, as demonstrated by lower relative standard deviation (RSD) values, which yield a lower LOD [137]. The use of these methods will help to minimize non-specific interactions, increasing the possibility of obtaining reliable results [32]. One case of non-specific binding, protein adsorption, may also be diminished by the same methods. Protein adsorption is also manifested through the appearance of autofluorescence (signal expansion), which, in addition to the above methods, can be avoided by coating substrates, which would inhibit non-specific interactions [140,141]. Polyelectrolyte multilayers (PEMs) are widely used in the detection process to reduce the non-specific adsorption of the proteins [141]. Examples of such multilayers are multilayers composed of poly(vinylpyrrolidone)/poly(acrylic acid) and heparin/chitosan. Such compounds have antibacterial properties and can reduce adhesion and destroy the contact properties [141]. Yu’s group presented a technique to reduce protein adsorption via performing stealth surface modification on SERS-active substrates [142]. Modifications such as these allow for the provision of nonfouling properties, which can decrease the non-specific adsorption of the protein and enable the performance of sensitive quantitative analyses of the molecules of interest [142]. Environmental sensitivity is also a common disadvantage of these methods, since the analysis process in both of these methods requires special temperature conditions and humidity levels, and special attention must be paid to the pH of the samples, as well as to the presence of water traces [143]. These factors may affect the risk of obtaining unreliable results and lead to the appearance of errors, and therefore, special attention should be paid to adjusting these parameters [144]. Another challenge of analysis through the use of SERS and fluorescent detection is the limited sensitivity to certain analytes that are unable to show strong signals on the spectra. This property of a certain kind of analytes significantly narrows the areas of analysis in which SERS and fluorescent detection methods can be applied. The uses of SERS and fluorescent detection are also problematic in the analysis of complex compounds, as well as in the simultaneous analysis of small analytes, since signal overlaps are possible [145]. Sandwich immunoassays with SERS detection provided additional signal enhancement due to the large number of Raman active reporter molecules (ten thousands) per single nanotag particle, which may correspond to a simple analyte molecule between the capture antibody on the substrate and the detection antibody at the surface of the nanotag gold particle [137].

### 3.2. Challenges and Solutions for Fluorescence Based Detection

The clinical application of the fluorescent LFIA method has been highlighted in the detection of SARS-CoV-2 biomarkers by Han et al. The sensitivity of colloidal Au-based LFIA performance was comparatively low and had a poor quantitative ability [52]. Fluorescent microspheres, carbon-based nanoparticles (NPs), SERS nanomaterials, and up-converting phosphors were used as signal markers to overcome the limitations of Au-based LFIA detection: quantum dots (QDs) and quantum dot beads were applied to LFIA. They demonstrated higher fluorescence sensitivity, in spite of their relatively small particle size and the requirement of relatively high light emission [52]. In their work, Han et al. attempted to design colorimetric fluorescent SiO_2_@Au/QD NPs with dual functionality to improve the sensitivity of detection [52]. Ao et al. observed the emission of optical labels only within the visible spectrum (400–700 nm) when they used those fluorophores in order to improve fluorescent LFIA sensitivity [63]. In their article, the autofluorescence of the quantum dots from various substances decreased the signal-to-noise ratio and interfered with the detection of low-concentrated analytes [63].

The fluorescent enzyme-linked immunosorbent assay was proposed as a substitute for the colorimetric ELISA, the limitations in detection of which are characterized by low sensitivity, low tolerance to matrix interferences, and differences in assay conditions [71]. Luo et al. also underlined the use of semiconductor QDs due to their enhanced photostability and higher quantum yields. However, the use of QDs in FIA as enzymatic labels lowers the activity of antibodies due to the random assignment of these QDs to their surface [50]. Another challenge noted for QD-based fluorescent immunoassays is the time-consuming purification process, owing to the QD’s small size [50]. Dong et al. highlighted the difficulty in synthesizing QD probes for fluorescent immunoassays with the use of heavy metals such as cadmium and lead [41].

Despite the high quantum yields provided by the QDs, fluorescence sensitivity remains an issue to be resolved. For low-concentrated analytes, the emission of the fluorescent signal is weak and interferes with the background noise [146]. As cited in Table 7, Roth et al. proposed a solution to reduce the autofluorescence of the magnetic bead, limiting the analytical detection performance of the fluorescent immunoassays by overlapping the fluorescent dyes and reducing the autofluorescence to 1% of its initial value. For the IL-8 immunoassay, photobleaching improved the limit of detection (LOD) from 0.017 to 0.006 ng/L (2.01 × 10^−15^ M to 7.10 × 10^−16^ M).

**Figure 5:**
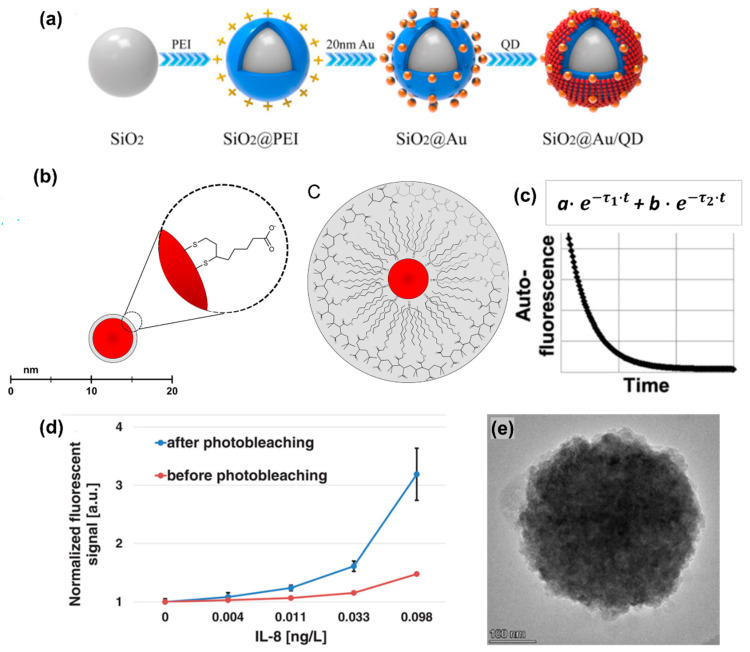
The solutions for fluorescence-based immunoassays used to improve the detections. (**a**) The schematic representation of how dual-functional SiO_2_@Au/QD fluorescent labels are fabricated. Adopted with modifications from Han et al. [52] Copyright © 2022 Elsevier B.V. (**b**) The scheme of QD operation by the ligand exchange, with dihydrolipoic acid on the left, and the QD coated with an amphiphilic polymer. Adopted with modifications from Esteve-Turrillas et al. [147] Copyright © 2013 Elsevier B.V. (**c**) The two-exponential model for fitting the photobleaching decay curve. Adopted with modifications from Roth et al. [146] Copyright © 2019 Elsevier B.V. (**d**) The graph of the measured LOD of the Interleukin-8 (IL-8) immunoassay with the dose–response curves, red and blue colors indicating the before and after of photobleaching the beads, respectively. Reused with modifications from Roth et al. [146] Copyright © 2019 Elsevier B.V. (**e**) The TEM image for the CDs@CaCO3 nanocomposite. Reused from Dong et al. [41] Copyright © 2021 Elsevier B.V. All permissions to use copyrighted images were obtained.

Another solution for sensitivity enhancement was proposed by Zhang et al., who introduced the fluorescence nanomaterial—upconversion nanoparticles. In comparison with traditional materials, these are characterized by low toxicity, high chemical stability, non-autofluorescence, high resistance to photobleaching, and large stoke shifts [54]. As for the toxicity associated with the synthesis of QD probes, Dong et al. proposed the use of carbon dots (CDs), with pronounced low costs, preparation simplicity, good photostability, and lower toxicity [41]. Similarly, Esteve-Turrillas et al. highlighted the lower toxicity levels for the large-sized QDs that used a polymer coating rather than small-ligand coatings, as shown in Figure 5b [147].

There is an increasing amount of scientific attention being directed towards the use of carbon dots, which were introduced as an alternative to quantum dots owing to their lower toxicity, and their potential use in biosensing and the detection of bacteria and biomarkers such as human immunoglobulin G [148,149]. The optical and imaging properties of the carbon dots showed relatively encouraging performances, with improved quantum yield, lower costs, and a simplified operation [150]. CDs’ desirable biocompatibility allows for their application in in vivo and in vitro cell imaging, as well as in the detection of cancer biomarkers [151]. Sultangaziyev et al. noted the significant fluorescence enhancement in single *E. coli* bacteria cells labeled with composite CdSeS/ZnS, with a general tendency towards the enhancement factor being reversely proportional to cyto-toxicity [18,152]. The measurements of the contrast ratios at both 532 and 633 nm excitation were used (1.0–1.2) to explain the unsatisfactory performance of MEF in labeling bacteria cells, due to the toxicity of CdTe QDs [152].

### 3.3. Challenges and Solutions for SERS IA Detection

Despite the many advantages of SERS, which include high sensitivity, efficient spectroscopic encoding, and lack of sensitivity to photobleaching and quenching, SERS immunoassay detection also faces a number of challenges [20]. Such disadvantages include difficulty in instrumentation, difficulty in performing dynamic analyses, the inconsistency of analysis speed for real-time analysis, as well as difficulties in obtaining reproducible signals [20]. Challenges in the application of SERS-based immunoassay detection and their possible solutions are illustrated in Table 8.

The problem with instrumentation is based on the cost of the analysis, since expensive substrates are often required for analysis. The solution to this problem may be found by using alternative substrates such as aluminum or silica. Bukasov’s group performed research, according to which substrates coated with these compounds can also provide highly sensitive results in the detection of proteins in biological fluid, especially in urea [18]. To obtain time-dependent information about analytes using SERS-based IA detection, it is necessary to carry out a number of repeated measurements at different time intervals. These measurements would enable a dynamic analysis of analytes with approximately similar levels of sensitivity. In addition, SERS has a relatively low speed of analysis and readout, which makes it difficult to conduct a real-time analysis using SERS-based IA detection. Another major difficulty associated with SERS is the reproducibility of the signals obtained as a result of the analysis, since the samples lose their stability when they are released in the form of nanoprobes, as many factors may affect the stability of samples, including environmental conditions (temperature, humidity, pH) [154]. Sometimes, rapid measurement is beneficial, especially in the analysis of biological molecules in which certain dynamic processes occur regularly. SERS-based IA detection has a relatively low throughput, which means that the time of measurement is usually longer for SERS than for fluorescence. As was mentioned in the Zong et al. study, a new wide-field Raman microscopy method has been developed to eliminate this problem [154].

**Figure 6:**
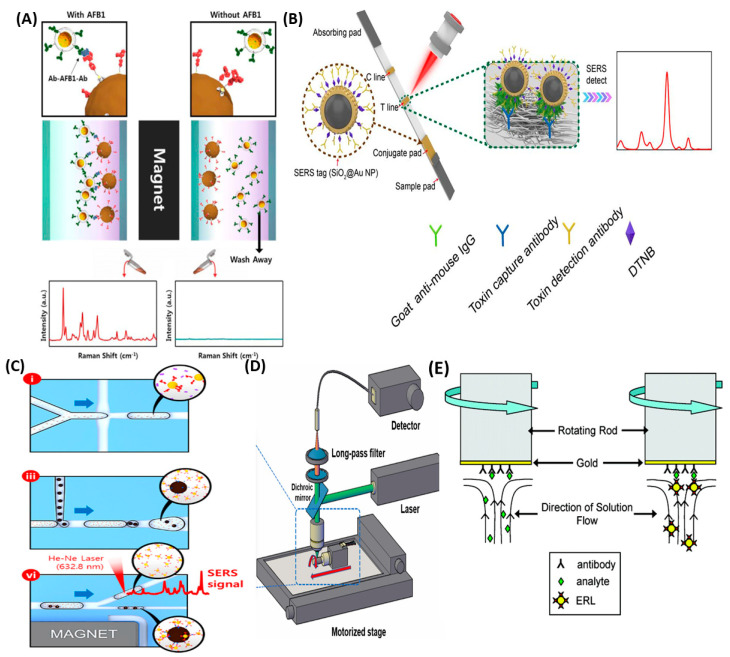
Alternative substrates used in SERS. (**A**) Scheme of SERS immunoassay SEHGNs and magnetic beads-based sensors for AFB1 detection. Adopted with modification from Ko et al. [108] © 2014 Elsevier B.V. (**B**) Representation of detection procedure of toxin-specific SERS–LFIA strip bases on SiO_2_@AuNPs. Adopted with modification from Jia et al. [95] © 2022 Elsevier Inc. (**C**) Images of the microdroplet compartments of the integrated SERS-based microfluidic channel, which illustrate: (i) droplet generation, (iii) droplet merging for the formation of magnetic immunocomplexes, (vi) Raman detection of unbound SERS nanotags in supernatant solution droplets. Adopted with modification from Choi et al. [91] **©** 2017 American Chemical Society. (**D**) Schematic representation of spiral scanning spectrometry. Adopted from Won Song et al. [155] © 2021, Elsevier. (**E**) Scheme of rotation substrate in analyte capture (left) and ERL labeling (right). Adopted from Driskell et al. [153] © 2007, American Chemical Society.

Furthermore, various non-stagnant substrates can be used to speed up analysis. In stagnant SERS sandwich immunoassays, the measurement time for an assay could reach 16–24 h, whereas some modern publications that use lateral flow immunoassays require less than 30 min. Figure 6 displays the varieties of substrates and other methods that make it possible to detect analytes quickly and productively using SERS-based IA.

A schematic representation of the SEHGNs and magnetic beads-based sensor is presented in Figure 6A [108]. Ko et al. presented a sensor for the detection of aflatoxin 1 (AFB 1) with an LOD of 3.2 × 10^−10^ M, and a detection time of less than 30 min. Anti-AFB1s and anti-ATB1 are two types of antibodies that were used to form this sandwich immunocomplex for the detection of AFB1. In the presence of an analyte, these anti-AFB1-conjugated SEHGNs and ATB1-conjugated magnetic beads form an antibody–antigen–antibody sandwich complex, and as can be seen in Figure 6A, a similar complex is not formed in the absence of an analyte (AFB1). Figure 6B shows the analysis process using toxin-specific SERS–LFIA strips based on SiO_2_@AuNPs [95]. These strips provide the opportunity to shorten the analysis time to 15 min, without impairing the method’s sensitivity. The LODs of this method for detecting ricin, SEB, and BoNT/A, respectively, were 1.54 × 10^−12^ M, 1.79 × 10^−12^, and 6.67 × 10^−12^ M. These analytes, in turn, are biological warfare agents, and due to the lethality of low doses of these compounds, rapid detection during their analysis is necessary. An approximate schematic representation of individual parts of the microdroplet channel filled with red ink is presented in Figure 6C [91]. This device was created for the detection of *Yersinia pestis* F1 antigen and is quite sensitive, since the LOD of this method is 3.85 × 10^−12^ M. According to more detailed diagrams, it is possible to note the parts of the microdroplet channel that are responsible for droplet generation, droplet merging, and droplet splitting. Thanks to the use of this multifunctional microfluidic platform, the detection time was reduced to 10 min. This method allows for efficient immunoreactions and wash-free detection, and can also be used for the detection of hazardous materials, since the analysis is carried out automatically in an isolated platform system. Spiral scanning spectrometry is another type of analysis that allows the user to speed up analysis time and improve the method’s sensitivity. As shown in Figure 6D, practically nothing has changed in terms of detection, and the main difference from the traditional method of analysis is that the substrate is attached to the rotor with double-sided tape [155]. During the analysis, the motor rotates the substrate at a speed of 600 rpm, allowing the entire area of the substrate to be analyzed. This method was used to reduce the optical damage, so that the damage done to the sample in such an analysis is less than in conventional types of analyses. This method can be used for the rapid quantitative analysis of hazardous or toxic compounds. Figure 6E illustrates the application of the rotating substrate method in the preparation of immunoassay samples for SERS detection by Porter’s group. In this method, a gold film-coated substrate is modified with linker molecules, such as dithiobis (succinimidyl propionate) (DSP). Then, the substrate is mounted on a rotating rod, modified with capture antibody, and blocked with a blocking buffer. Each step occurs within several minutes, not several hours, as occurs in a typical stagnant assay. When the rod with the substrate attached to it starts to rotate at the next step, an antigen/analyte (the rabbit IgG) would diffuse quickly to the capture substrate and would bind to antigen. Finally, at the last stage before drying, the substrate is washed in the ERL (nanotag) solution, while being rotated on a rod at a controlled speed. This method allows for a significant reduction in the immunoassay time from more than several hours to 25 min, without any decrease in its sensitivity, and a 10+ fold improvement in efficiency. In addition to a large improvement in assay time, the reported rotating substrate procedure reduced non-specific binding, and therefore improved the assay’s selectivity [153].

## 4. Graphical Comparison of FOMs in Analytical and Clinical Assays (SERS vs. Fluorescence)

**Figure 7:**
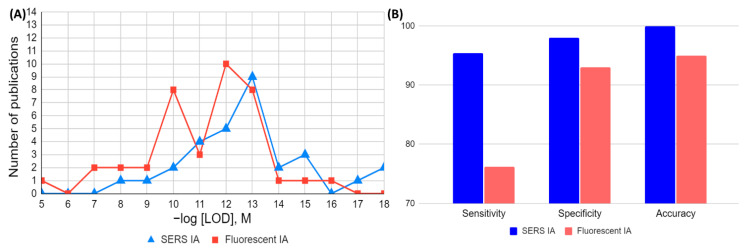
Graphical comparison of (**A**) analytical (LOD) and (**B**) clinical Figures of Merit (FoM) for immunoassays with SERS and fluorescence detection (FIA).

Finally, the graphical comparison of FOMs reported in analytical and clinical IAs with fluorescence-based and SERS-based detection is demonstrated in Figure 7.

The 30 analytical papers per detection method (30 for SERS and 30 for fluorescence) were used to calculate the median and geometric mean of the LOD values. The distribution of these 60 analytical publications by −log[LOD] is shown in Figure 7A. The comparison demonstrates that assays that use SERS detection are generally more sensitive, since their median and geometric mean for LOD were 4.3 × 10^−13^ M and 5.4 × 10^−13^ M, respectively, as compared to 1.5 × 10^−11^ M and 2.8 × 10^−11^ M for fluorescent methods. There are eight ultra-sensitive reports (LOD ≤ 10 fM) for SERS IAs and only three ultrasensitive reports, with LOD ≤ 10 fM (rounded up), for IAs with fluorescence-based detection.

Figure 7B is based on median clinical FOMs, calculated and shown in Table 4. Figure 7B demonstrates that SERS detection showed higher sensitivity, specificity, and accuracy, in comparison with IAs using fluorescence detection. For instance, sensitivity has a median value of 96% for SERS and 76% for fluorescence based IAs. The median value of specificity is 98% for clinical SERS IAs and 93% for clinical fluorescence-based IAs, and the same trend is observed for median accuracy.

## 5. Conclusions

Overall, after we converted the reported LODs into units of molarity of the same concentration, when necessary, we observed that SERS IA methods have a sensitivity (LOD) 1.5 to 2 orders of magnitude (on average) greater in comparison to fluorescence-based IAs.

As mentioned above, the median and average values for major clinical FOMs reported in the literature are substantially higher for SERS IAs in comparison to those values for fluorescence IAs.

Comparing the reproducibility of SERS vs. fluorescence IAs, when RSD values are reported, we observe no significant difference in average RSD (5–6%), and therefore no significant difference in reproducibility. The median LODs we calculated for the same group of reports was one order of magnitude lower for SERS IAs than for fluorescence-based IAs.

Both SERS and fluorescence immunoassay detection face several common challenges, such as non-specific binding and protein adsorption, which decrease reproducibility, selectivity, and sensitivity in analyte detection. Another more fluorescence-specific problem is photobleaching, which is also considered in this review. Solutions to this include the application of mixed SAMs on a substrate and/or sometimes the application of non-noble metal substrates (Al, Si, etc.), the application of high-quality capture antibodies, and the application of rotating substrates, which would decrease both non-specific protein adsorption and assay time. Finally, a way to minimize photobleaching in fluorescence-based assays is to use preferentially non-toxic quantum dots, including carbon dots. Since immunoassays with fluorescence are already commercialized as a detection method, we hope that the development of immunoassays with SERS detection will attract more attention in the scientific and clinical research community, and would lead to the commercialization of SERS-based immunoassays as well. For instance, a decrease in IA time can be driven by the application of rotating substrates, which can be combined with developments in mixed monolayers on the substrate and/or on nanotags, aimed to decrease non-specific binding. Both of these may significantly improve the applicability of SERS-based immunoassays, promoting the commercial application of these assays. Based on these or other developments, which may include, for instance, SERS, LFIA, or SERS IA with magnetic beads, we would see more successful reports on the multiplexing detection of biomarkers in human or animal bio fluids (blood, urine, saliva). Eventually, greater knowledge will lead to better quality, and startups/companies may start seeking and acquiring approval from regulatory organizations (FDA, etc.), for instance, to start applying SERS IAs in noninvasive clinical diagnostics. Eventually, SERS IAs may finally be adopted as a commercial analytical method for use in clinical diagnostics.

## Figures and Tables

**Table 1:** Analytical performance parameters for fluorescence immunoassay-based detections.

Year, Author	Analytical Technique	Analyte	LOD (MW, kDa)	LOD, M	Other FoM (Linear Range (LR)/Dynamic Range (DR), Recovery, R^2^)
2022, Wang [35]	FI based on PPi with MOFs	chloramphenicol (amide alcohols antibiotic)	0.028 μg/L, 0.323	8.7 × 10^−8^	LR 0.05–0.75 μg/L; 91.8–112.3%; -; 0.99
2021, Sun [36]	FI (silver-amplified)	α-fetoprotein (cancer biomarker)	42 pg/mL, 66.478	6.3 × 10^−13^	LR 0.1–5 μg/mL; -; 0.999
2021, Luo [37]	Fl	acrylamide food contamination)	0.16 μg/L, 0.0718	2.4 × 10^−9^	LR 0.21–6.48 μg/L; 82.3–105.6%; 0.988
2017, Wang [38]	Fl	α-fetoprotein (cancer biomarker)	0.45 ng/mL, 66.478	6.8 × 10^−12^	LR 1–80 ng/mL; -; 0.994
2022, Chen [39]	Dual-mode Fl (with colorimetry)	ochratoxin A (mycotoxin)	0.17 ng/L, 0.404	4.2 × 10^−13^	LR 0.001–10 μg/L; 95.0%− 103.8; 0.995
2018, Chen [40]	ELISA	α-fetoprotein (cancer biomarker)	0.041 ng/mL, 66.478	6.2 × 10^−13^	LR 0.2–1 ng/mL; -; 0.971
2018, Chen [40]	ELISA	α-fetoprotein (cancer biomarker)	0.041 ng/mL, 66.478	6.2 × 10^−13^	LR 1–4 ng/mL; -; 0.995
2021, Dong [41]	FI	chloramphenicol (amide alcohols antibiotic)	0.03 μg/kg, 0.323	9.3 × 10^−11^	LR 0–10 μg/kg; 83.7–105.0%; 0.993; assay time 30 min
2017, Xie [42]	FI	IgG (against bacterial infections)	0.006 ng/mL, 150	4.0 × 10^−14^	LR 0.01–800 ng/mL; -; -
2014, Li [43]	Surface-Enhanced Fluorescence (SEF) immunosensor	microcystin-LR (food contamination)	0.007 ng/mL, 0.995	7.0 × 10^−12^	LR 0.02–16 ng/mL 98.0–102.2%; 0.9981
2021, Liu [44]	Horseradish Peroxidase (HRP)-ELISA	zearalenone (mycotoxin)	0.017 ng/mL, 0.318	5.3 × 10^−11^	LR 0.02–0.625 ng/mL; 95.56–103.33%; 0.996; RSD = 1.0–8.39%
2019, Zhu [45]	ELISA	sulfamethazine (against bacterial infections)	0.05 μg/L, 0.278	1.8 × 10^−7^	DR 0.1400–71.71 μg/L; 84.18–118.6%; -
2016, Lv [46]	Paper-based analytical device (PAD) based FI	carcinoembryonic antigen (cancer marker)	0.041 ng/mL, 180	2.3 × 10^−13^	DR 0.1–200 ng/mL
2019, Zhao [47]	Alkaline Phosphatase (ALP)-based FI	α-fetoprotein (cancer biomarker)	0.21 ng/mL, 66.478	3.2 × 10^−12^	LR 0.5–40 ng/mL; -; 0.997
2016, Chen [48]	Catalase (CAT) -based ELISA	*E. coli* (bacteria detection)	5 × 10^2^ CFU/mL		DR 5–5 × 10^6^ CFU/mL; 65.88–105.6%; 0.9925
2019, Li [49]	FIA	imidacloprid (pesticide residues screening)	0.1 ng/mL, 0.256	3.9 × 10^−10^	DR 0.1–50 ng/mL; 85.4–107.4%; 0.990
2018, Luo [50]	RF-ELISA	ethyl carbamate (food safety monitoring)	2.6 μg/L, 0.891	2.9 × 10^−5^	LR 3.9–105.0 μg/L; 86.0∼102.70%; -; CV = less than 11.6%
2020, Liu [51]	ALP-based FI	alkaline phosphatase (liver damage or bone disorder marker)	0.1 mU/mL, 86		LR 0.1–6 mU/mL; 100.2–101.8%; 0.998; RSD = less than 5%
2020, Liu [51]	ALP-based FI	cardiac troponin I (cardiac marker)	1 ng/mL, 24	4.2 × 10^−11^	LR 1–30 ng/mL; -; 0.997
2020, Liu [51]	ALP-based FI	cardiac troponin I (cardiac marker)	1 ng/mL, 24	4.2 × 10^−11^	LR 30–250 ng/mL; -; 0.992
2022, Han [52]	LFIA	S1 protein of SARS-CoV-2 (COVID-19 marker)	0.033 ng/mL, 76.9	4.3 × 10^−13^	DR 0.05–1000 ng/mL; -; -; RSD = 6.89–7.16%
2021, Pan [53]	Indirect competitive enzyme-linked immunosorbent assay (ic-ELISA)	Dicofol (food safety monitoring)	0.62 ng/mL, 0.371	1.7 × 10^−12^	LR 1.36–19.92 ng/mL; -; 0.985
2020, Zhang [54]	Upconversion Nanoparticles (UCNPs)-based FIA	tyramine (food safety monitoring)	0.1 μg/L, 0.131	7.6 × 10^−7^	LR 0.5–100 μg/L; -; 0.9988
2020, Zhang [54]	UCNPs-based FIA	histamine (food safety monitoring)	0.01 μg/L, 0.111	9.0 × 10^−8^	LR 0.1–100 μg/L; -; 0.9954
2022, Wang [55]	LFIA with Polydopamine Nanospheres (PDANs)	sulfamethazine (against bacterial infections)	0.043 ng/mL, 0.278	1.5 × 10^−13^	LR 0.05–10 ng/mL; 88.9–105.9%; 0.9910
2020, Wang [56]	PT-FIA	ochratoxin A (mycotoxin)	0.12 ng/mL, 0.404	3.0 × 10^−13^	LR 0.2–1.26 ng/mL; -;-
2020, Zhang [57]	Multi-analyte FIA	triazophos (organophosphate pesticides monitoring)	0.007 μg/L, 0.313	2.2 × 10^−11^	LR 0.01–20 μg/L; 77.7–113.6%; 7.1–17.1%
2020, Zhang [57]	Multi-analyte FIA	parathion (organophosphate pesticides monitoring)	0.009 μg/L, 0.291	3.1 × 10^−11^	LR 0.05–50 μg/L; -; -
2020, Zhang [57]	Multi-analyte FIA	chlorpyrifos (organophosphate pesticides monitoring)	0.087 μg/L, 0.351	2.5 × 10^−10^	LR 0.5–1000 μg/L; -; -
2020, Lin [58]	Microfluidic fluorescence immunoassay	procalcitonin (infectious disease biomarker)	0.05 ng/mL, 13	3.9 × 10^−12^	DR 0.10–70.00 ng/mL; -; 0.994; detection time 10 min
2019, Li [59]	FI	bromadiolone (Rodenticides detection)	0.047 ng/mL, 0.527	8.9 × 10^−11^	LR 0.11–3.09 ng/mL; 77.9–85.6%; 0.987
2021, Nishiyama [60]	FPIA	c-reactive protein (marker of inflammation)	1.58 μg/mL, 120	1.3 × 10^−8^	DR 5–20 μg/mL; -; -; detection time 10 min
2021, Shokri [61]	FAIA	CP25 protein (Citrus Tristeza virus detection)	220 pg/mL, 0.620	3.6 × 10^−10^	LR 0.4–25 ng/mL; -; 0.995
2020, Su [62]	FCMFI	aflatoxin B1 (mycotoxin)	0.2034 μg/L, 0.312	6.5 × 10^−10^	LR 0–3 μg/L; -; 0.9642; LOQ = 0.5659 μg/L
2022, Ao [63]	LFIA	carcino-embryonic antigen (cancer marker)	0.11 ng/mL, 180	6.1 × 10^−13^	LR 0.11–100 ng/mL; -; -
2022, Ao [63]	LFIA	cytokeratin 19 fragment (cancer biomarker)	0.18 ng/mL, 40	4.5 × 10^−12^	LR 0.18–100 ng/mL; -; -
2022, Ao [63]	LFIA	neuron-specific enolase (cancer biomarker)	0.28 ng/mL, 78	3.6 × 10^−12^	LR 0.28–100 ng/mL; -; -
2020, Othman [64]	NCD-based FI	nuclear matrix protein 22 (bladder cancer biomarker)	0.047 ng/mL		LR 1.3–16.3 ng/mL; 96.50–103.61%; 0.99; RSD = 0.90–4.85%
2021, Guo [65]	FRET immunoassay	GO-imidaclothiz antigen (food safety monitoring)	0.08 ng/mL, 0.262	3.1 × 10^−10^	DR 0.08–50 ng/mL; -; 0.9934
2016, Li [66]	FRET immunoassay	BaP (environmental pollutant)	0.06 ng/mL, 0.225	2.7 × 10^−10^	LR 0.1–5 ng/mL; 80.5–87.0% and 92.9–92.1%; 0.9929
2020, Lee [67]	Single membrane-based LFIA	cardiac troponin I (cTnI) (cardiac marker)	97 pg/mL, 24	4.0 × 10^−12^	LR 0–1.16 ng/mL; -; 0.99;
2021, Yang [68]	SERS/FI	carcinoembryonic antigen (cancer marker)	0.1 pg/mL, 180	5.6 × 10^−16^	LR 0.5–400 pg/mL; 105.50–106.73%; 0.9910
2019, Xie [69]	FI	aflatoxin B1 (mycotoxin)	0.002 ng/mL, 0.312	6.4 × 10^−12^	LR 0.01–0.5 ng/mL; 96.5–119.0% and 98.0–135.0%; 0.995;
2018, Zvereva [70]	FPIA	Colchicine (inflammation marker?)	1.8 ng/mL, 0.399	4.5 × 10^−9^	DR 4.1–74.3 ng/mL; 79–108%; -; detection time 10 min
2014, Wang [71]	TRFIA	diethylstilbestrol (environmental pollutant)	0.4 fg/mL, 0.268	1.5 × 10^−15^	LR 1.0 × 10^−6^–10 ng/mL; 88–105%; 0.9989
2019, Dong [72]	ELISA	Amantadine (food contamination)	0.035 ng/mL, 0.151	2.3 × 10^−10^	LR 0.048–1.1 ng/mL; 72.6%-90.4%; -
2010, Sun [73]	FIA	17β-oestradiol (environmental estrogen pollution)	0.00542 ng/mL, 0.272	2.0 × 10^−10^	LR 0.01–10,000 ng/mL; 86–113%; 0.9887
2021, Tian [74]	FPIA	α-fetoprotein (cancer biomarker)	0.28 ng/mL, 66.478	4.2 × 10^−12^	LR 0.5–500 ng/mL; 97.0–107.4%; 0.993;
		carcinoembryonic antigen (cancer marker)	0.36 ng/mL, 180	2.0 × 10^−12^	LR 0.5–500 ng/mL; 93.0–104%; 0.996;
			LOD Median, M	1.5 × 10^−11^	
			Geometrical mean, M	2.8 × 10^−11^	

Abbreviations: FoM—Figure of Merit, MW—molecular weight, LOD—limit of detection, LR—Linear Range, DR—Dynamic Range, LDR—Linear Dynamic Range, FIA—fluorescent immunoassay, ELISA—enzyme-linked immunosorbent assay, LFIA—lateral flow immunoassay, FRET—fluorescence energy transfer, FCMFI—flow cytometry fluorescence immunoassay, RF-ELISA—ratiometric fluorescence enzyme-linked immunosorbent assay, TR-FRET—time-resolved fluorescence energy transfer, TRFIA—time-resolved fluorescence immunoassay, FPIA—fluorescence polarization immunoassay, FAIA—Fluorescence Anisotropic Immunoassay, NCD-based FI—Nitrogen-Doped Carbon Dots-based Fluorescence Immunoassay, R^2^—coefficient of determination, “-“ information is not available.

**Table 2:** The analytical FoMs of SERS-based immunoassay detection of analytes.

Year, Authors	Analytical Technique (Objective, Excitation Laser, Assay Type)	Analyte	LOD (MW, kDa)	LOD (M)	Other FoM (Linear Range (LR)/Dynamic Range (DR)/Linear Dynamic Range (LDR), Recovery, RSD)
2021, Qu [75]	SERS (-, 633 nm, Stagnant on solid substrate)	HIgG (human immunoglobulin G)	0.1 μg/mL (150)	6.7 × 10^−10^	LR 0.1–200 μg/mL; 103.6–105.3%; 4.55–4.66%
2013, Baniukevic [76]	SERS (20×, 785 nm, in solution)	gp51 (bovine leukemia virus antigen)	0.95 μg/mL (51)	1.9 × 10^−8^	LR 0–0.06 mg/mL; 85.5–100%; 7.31–8.9%; LOQ 3.14 μg/mL; R^2^ = 0.9983
2019, Xiao [77]	SERS (-, 785 nm, LFIA)	H7N9 (avian influenza virus)	0.0018 HAU		LR 0.0025–0.5 HAU; -; -; detection time 20 min
2018, Shi [78]	SERS (50×, 632.8 nm, LFIA)	NEO (neomycin) (broad spectrum antibiotic)	0.216 pg/mL (0.615)	3.5 × 10^−13^	-; 89.7–105.6%; 2.4–5.3% (n = 3); IC50 0.04 ng/mL
2020, Achadu [79]	SERS (sandwich-type immunoassay) (100⨯, 785 nm, Stagnant on solid substrate)	norovirus (NoV) (cause of gastritis and colitis)	5.2 fg/mL		LR 10 fg/mL–100 ng/mL; -;7.95–8.67%
2020, Panikar [80]	SERS (50×, 785 nm, Stagnant on solid substrate)	B7-H6 (tumor biomarker)	10.8 fg/mL	1.0 × 10^−13^ M	NS 10^−10^–10^−14^ M; -; 4.8%
2018, Bozkurt [81]	SERS (-, 785 nm, in solution)	*E. coli* (Bacteria detection)	10 cfu/mL		LR 1.7 × 10^1^–1.7 × 10^6^ cfu/mL; -; -; R^2^ = 0.992
2016, She [82]	SERS (-, 785 nm, Stagnant on solid substrate)	Hg^2+^ (hazardous pollutants in the environment)	0.45 pg/mL (0.201)	2.2 × 10^−12^	DR 10^−3^–100 ng/mL; 88.3–107.3%; 1.5–9.5% (n = 3); IC50 0.12 ng/mL;
2019, Ilhan [83]	SERS (10×, 785 nm, LFIA)	*E. coli* (Bacteria detection)	0.52 cfu/mL		LR 10^1^–10^7^ cfu/mL; -; -; R^2^ = 0.984; LOQ = 1.57 cfu/mL
2020, Du [84]	SERS (-, 785 nm, Stagnant on solid substrate)	PSA (prostate cancer biomarker)	1.871 pg/mL (27.76)	6.7 × 10^−14^	LR 10^−4^–10^−12^ g/mL; -; -; R^2^ = 0.987
2021, Wang [85]	SERS (-, 785 nm, Stagnant on solid substrate)	cardiac troponin I (cTnI) (cardiac marker)	3.16 pg/mL (24)	1.3 × 10^−13^	LR 0.01–100 ng/mL; 94.9–121.6%; -; CV below 15%
2021, Wang [85]	SERS (-, 785 nm, Stagnant on solid substrate)	creatine kinase isoenzyme MB (CK-MB) (cardiac marker)	4.27 pg/mL (87)	4.9 × 10^−14^	LR 0.01–100 ng/mL; 94.9–121.6%; -; CV below 15%
2016, Chang [86]	SERS (-, 532 nm, Stagnant on solid substrate)	PSA (prostate cancer biomarker)	3.4 fM (27.76)	3.4 × 10^−15^	DR 0.001–1000 ng/mL; -; -
2019, Kim [87]	SERS (20×, 633 nm, in solution)	botulinum neurotoxins (BoNTs) Type A (antitoxin)	5.7 ng/mL (150)	3.8 × 10^−11^	DR 0 ng/mL–1.0 μg/mL; -; -; assay time less than 2 h
2019, Kim [87]	SERS (20×, 633 nm, in solution)	botulinum neurotoxins (BoNTs) Type B (antitoxin)	1.3 ng/mL (159)	8.5 × 10^−12^	DR 0 ng/mL–1.0 μg/mL; -; -; assay time less than 2 h
2022, Kim [88]	SERS (-, 638 nm; 725 nm, Stagnant on solid substrate)	adiponectin (Gestational diabetes mellitus (GDM) biomarker)	3.0 × 10–16 g/mL (30)	1.0 × 10^−17^	NS 10^–15^–10^–6^ g/mL; -; -; R^2^ = 0.994;
2019, Gao [89]	SERS (10×, 632.8 nm, in solution)	PSA (prostate cancer biomarker)	0.01 ng/mL (27.76)	3.6 × 10^−13^	LR 0.01–100 ng/mL -; -
2014, Song [90]	SERS (-, 785 nm, Stagnant on solid substrate)	HIgG (defending against viruses or bacteria)	1 fg/mL (150)	6.7 × 10^−18^	LR 1 fg/mL-1ng/mL; -; -
2017, Choi [91]	SERS (20×, 632.8 nm, on substrate, in solution)	F1 antigen for *Yersinia pestis* (biological weapon)	59.6 pg/mL (15.5)	3.9 × 10^−12^	-; -; -; R^2^ = 0.959; assay time 10 min
2019, Yang [92]	SERS (10×, 633 nm, Stagnant on solid substrate)	AFP (liver, ovaries, testicles cancer biomarker)	0.3 fg/mL (67.5)	4.4 × 10^−18^	LR 1 fg/mL–1 ng/mL; 94.36–102.12%; 8.21%
2021, Tian [74]	SERS (-, 785 nm, in solution)	CA 19-9 (pancreatic cancer biomarker)	5.65 × 10^−4^ IU/mL		LR 0.001–1000 IU/mL; -; 5.65%
2019, Huang [93]	SERS (-, 785 nm, Stagnant on solid substrate)	albumin (diabetic nephropathy and cardiovascular disease marker)	0.2 mg/L (66.5)	3.0 × 10^−9^	LR 10–300 mg/L; -; -
2016, Yang [94]	SERS (-, 632.8 nm, in solution)	chloramphenicol (CAP) (amide alcohols antibiotic)	1.0 pg/mL (0.323)	3.1 × 10^−12^	DR 1–1 × 10^4^ pg/mL; 81.42–96.92%; 9.7–14.4%
2022, Jia [95]	SERS (-, 785 nm, LFIA)	ricin (biological warfare agent)	0.1 ng/mL (65)	1.5 × 10^−12^	LDR 0.05–10^3^ ng/mL; -; <2.5%; detection time 15 min
2022, Jia [95]	SERS (-, 785 nm, LFIA)	SEB (biological warfare agent)	0.05 ng/mL (28)	1.8 × 10^−12^	LDR 0.05–10^3^ ng/mL; -; <4.75%; detection time 15 min
2022, Jia [95]	SERS (-, 785 nm, LFIA)	BoNT/A (biological warfare agent)	1 ng/mL (150)	6.7 × 10^−12^	LDR 0.05–10^3^ ng/mL; -; <3.2%; detection time 15 min
2022, Gao [96]	SERS (50×, 632.8 nm, in solution)	creatine kinase MB isoenzyme (CK-MB) (cardiac marker)	7.92 pg/mL (87)	9.1 × 10^−14^	-; -; -
2022, Gao [96]	SERS (50×, 632.8 nm, in solution)	cardiac troponin (cTnI) (cardiac marker)	2.94 pg/mL (24)	1.2 × 10^−13^	-; -; -
2023, Kaladharan [97]	SERS (-, 785 nm, Stagnant on solid substrate)	SARS-CoV-2 spike protein (COVID-19 marker)	50 pg/mL		-; -; -; reaction time 20 min
2023, Kaladharan [97]	SERS (-, 785 nm, Stagnant on solid substrate)	SARS-CoV-2 virus-like-particle (VLP) (COVID-19 marker)	50 pg/mL (in PBS); 400 pg/mL (untreated saliva)		LR 1000 ng/mL–100 pg/mL; -; -; reaction time 20 min
2023, Chen [98]	SERS (-, 785 nm, LFIA)	aflatoxin B1 (AFB1) (mycotoxin)	0.24 pg/mL (0.312)	7.7 × 10^−13^	LDR 250 fg/mL–25 ng/mL; 91.0% ± 6.3–104.8% ± 5.6%; -
2023, Chen [98]	SERS (-, 785 nm, LFIA)	ochratoxin A (OTA) (mycotoxin)	0.37 pg/mL (0.404)	9.2 × 10^−13^	LDR 250 fg/mL–25 ng/mL; 87.0% ± 4.2–112.0% ± 3.3%; -
2022, Mohammadi [99]	SERS (-, 785 nm, in solution)	SARS-CoV-2 (spike protein) (COVID-19 marker)	4.7 fg/mL		-; -; -; R^2^ = 0.9932
2013, Granger [24]	SERS (-, 632.8 nm, Stagnant on solid substrate)	MMP-7 (cancer marker)	2.28 pg/mL	7.9 × 10^−14^	-; -; -; R^2^ = 0.989
2013, Granger [24]	SERS (-, 632.8 nm, Stagnant on solid substrate)	CA 19-9 (pancreatic cancer biomarker)	34.5 pg/mL	1.6 × 10^−13^	-; -; -; R^2^ = 0.991
2011, Zhu [100]	SERS (-, 632.8 nm, Stagnant on solid substrate)	Clenbuterol (drug for the pulmonary disease)	0.1 pg/mL (0.277)	3.6 × 10^−13^	DR 0.1–100 pg/mL; 96.9–116.5%; 9.1%
2019, Fu [101]	SERS (20×, 633 nm, in solution)	Cardiac troponin I (cTnI) (cardiac marker)	5 pg/mL	2.1 × 10^−13^ (24)	LR 0.01–1000 ng/mL; -; -
2023, Tuckmantel Bido [102]	SERS (50×, 632.8 nm, Stagnant on solid substrate)	SARS-CoV-2 spike protein (COVID-19 marker)	34.9 pM	3.5 × 10^−11^	-; -; -; LOQ = 105.7 pM
2023, Xie [103]	SERS (-, -, in solution)	IL-6 (ovarian cancer biomarker)	0.028 pg/mL (21)	1.3 × 10^−15^	LR 0.1–1000 pg/mL; 80–117%; 5.8–10.9%
2022, Yin [104]	SERS (-, 532, 638 and 785 nm, LFIA)	Zearalenone (ZEN) (mycotoxin)	3.6 μg/kg (0.318)		-; 86.06–111.23%; 8.45∼11.37%; coincidence rate 86.06–111.23%;
2017, Cheng [105]	SERS (20×, 633 nm, in solution)	free PSA (f-PSA) (prostate cancer biomarker)	0.012 ng/mL (27.76)	4.3 × 10^−13^	LR 4.0–10.0 ng/mL; -; -; assay time < 1 h
2017, Cheng [105]	SERS (20×, 633 nm, in solution)	complexed PSA (c-PSA) (prostate cancer biomarker)	0.15 ng/mL (27.76)	5.4 × 10^−12^	LR 4.0–10.0 ng/mL; -; -; assay time < 1 h
2022, Jiao [106]	SERS (-, 785 nm, Stagnant on solid substrate)	ochratoxin A (OTA) (mycotoxin)	2.46 pg/mL (0.404)	6.1 × 10^−12^	LR 0.001–10 ng/mL; 77.68–104.69%; -; assay time 90 min
2022, Jiao [106]	SERS (-, 785 nm, Stagnant on solid substrate)	fumonisin B1 (FB1) (mycotoxin)	0.20 pg/mL (0.722)	2.8 × 10^−13^	LR 0.001–10 ng/mL; 76.39–117.73%; -; assay time 90 min
2022, Jiao [106]	SERS (-, 785 nm, Stagnant on solid substrate)	deoxynivalenol (DON) (mycotoxin)	68.98 pg/mL (0.296)	3.3 × 10^−11^	LR 0.1–1000 ng/mL; 70.95–113.16%; -; assay time 90 min
2022, Cha [107]	SERS (20×, 633 nm, in solution)	SARS-CoV-2 (COVID-19 marker)	2.56 fg/mL		-; -; -; detection time < 30 min
2015, Ko [108]	SERS (-, 633 nm, in solution)	aflatoxin B1 (AFB1) (mycotoxin)	0.1 ng/mL (0.312)	3.2 × 10^−10^	-; -; -; analysis time < 30 min
2017, Yang [109]	SERS (-, 532 nm, Stagnant on solid substrate)	α-fetoprotein (AFP) (liver, ovaries, testicles cancer biomarker)	0.081 pg/mL (67.5)	1.2 × 10^−15^	LR 0.5–100 pg/mL; -; 3.4–7.4%
2021, Yang [110]	SERS (50×, 785 nm, in solution)	amantadine (AMD) (broad-spectrum antiviral drug)	0.0038 μg/L (0.151)	2.5 × 10^−11^	LR 0.01–50 μg/L; 82.0–106.0%; 4.7–9.6%; detection time 30 min
2022, Liu [111]	SERS (-, 785 nm, LFIA)	acrosomal protein SP10 (sperm concentration detection biomarker)	25.12 fg/mL		LDR 100 fg/mL–10 ng/mL; 92.5–110.9%; 6%
2019, Deng [112]	SERS (-, 785 nm, stagnant on solid substrate)	pharmaceutical diclofenac (DCF) (non-steroidal anti-inflammatory drug)	0.07 pg/mL (0.334)	2.1 × 10^−13^	-; 92.5–106.0%; 4.5–5.7%; IC50 = 9 pg/mL; detection time 15 min;
		median (30), M	4.32 × 10^−13^		
		average (30), M	5.85 × 10^−10^		
		geometric mean (30), M	5.38 × 10^−13^		

Abbreviations: FoM—Figure of Merit, SERS—surface-enhanced Raman spectroscopy, LOD—limit of detection, LR—Linear Range, MW—molecular weight in kilo Daltons, DR—Dynamic Range, LDR—Linear Dynamic Range, LFIA—lateral flow immunoassay. R^2^—coefficient of determination, “-“ information is not available.

**Table 3:** Median and geometric mean LOD values for each type of assay of analytical papers on SERS immunoassays.

Type of Assay (Total Number of Works, Number of Works Involved in Calculations)	Median LOD, M	Geometric Mean of LOD, M
Stagnant on solid substrate (18, 16)	1.2 × 10^−13^	8.5 × 10^−14^
LFIA (7, 3)	1.2 × 10^−12^	1.3 × 10^−12^
In solution (15, 11)	3.1 × 10^−12^	2.6 × 10^−12^

Abbreviations: LFIA—lateral flow immunoassay, LOD—limit of detection.

**Table 4:** Clinical studies on SERS- and fluorescence-based immunoassays.

Year, Authors	Analytical Technique	Analyte	Sensitivity	Specificity	Accuracy	Number of Samples	Other FoM
2021, Liu [114]	SERS LFIA	IgG; IgM (defending against viruses or bacteria)		100%	100%	68 (19+; 49−)	AUC: IgM + IgG (1–0.997); IgM (0.997–0.941) and IgG alone (0.986–0.977)
2021, Banaei [115]	SERS	extracellular vesicles (EVs) (delivery of biomolecules to recipient cells)	95%	96%	-	15 (10+; 5−)	
2019, Zamora-Mendoza [116]	SERS	proteins (IL-8, IL-10, sCD163) (immunomodulatory cytokine)	85	82	84	44 (26+; 18−)	
2022, Li [117]	SERS	LRG1-positive exosomes (LRG1-Exos) and GPC1-positive exosomes (GPC1-Exos)	91.4%	86.7%	-	50 (15 normal, 35 tumor)	AUC of the 2-molecule panel 0.95; LOD 15 particles/µL
2020, Lu [118]	SERS LFIA	AFP (ovaries, testicles, liver cancer biomarker)		-	100	19 (+ − not given)	LOD 9.2 pg/mL; range 10 pg/mL-500 ng/mL
2018, Li [119]	SERS IA	pancreatic cancer derived exosomes	95.7	-	-	103 (71+, 32−)	LOD 9 × 10^−19^ M
2018, Jia [120]	SERS LFIA	human IgM (pathogens neutralization)	-	-	100	20 (all MP-specific IgM positive)	LOD 0.1 ng/mL; detection rate = 100%
2022, Lu [121]	SERS LFIA	Influenza (virus)	100	100	100	39(28 SARS-CoV-2 +; 6 influenza +; 5−)	LOD = 23 HAU/mL
		SARS-CoV-2 (COVID-19 marker)					LOD = 5.2 PFU/mL
2019, Lee [122]	SERS LFIA	*O. tsutsugamushi* IgG (defending against viruses or bacteria)	100	100	100	40 (16+; 24−)	-
		median, M (Number of publications)	95.35 (6)	98 (6)	100 (6)		
2019, Squire [123]	FIA	NT-proBNP (diagnostic screening)	65	93	78	40	range 0–100 pg/mL; R^2^ = 0.86
2021, Lu [124]	TF-LFIA	brucella antibody (infectious disease detection)	98.57	100	99.63		LOD = 0.3 IU/mL; LLOQ = 1.6 IU/mL; R^2^ = 0.9961
2019, Lee [122]	IFA	*O. tsutsugamushi* IgG (scrub typhus biomarker)	96	94	95	40 (16+; 24−)	
2015, Radon [125]	GeLC-MS/MS analysis, immunoassay—ELISA	LYVE-1, REG1A, and TFF1 (prostate cancer)	76.9%	86.8%	-	279 (192+, 87−)	
2017, Wang [126]	LC-MS, immunoassay—ELISA	Flotilin-2, PARK7 (Prostate cancer)	68%	93%		42 (26+, 16−)	
2019, Shimur [127]	Immunoassay—ELISA	uCyr61, uTFF3, (Colorectal cancer)	75.5%	69.8%		258 (148+, 110−)	
2016, Gomez [128]	FIA	Influenza A (respiratory virus)	75.3%	98.6%		1057	
2016, Gomez [128]	FIA	Influenza B (respiratory virus)	50.0%	92.4%		1057	
2016, Gomez [128]	FIA	RSV (respiratory virus)	92.1%	91.8%		261	
2021, Daag [129]	Immunoglobulin fluorescence immunoassay	Dengue serostatus (dengue fever detection)	95%	98%		1000	
		median, M (number of publications)	76.2 (8)	93 (8)	95 (3)		

Abbreviations: FoM—Figure of Merit, SERS—surface-enhanced Raman spectroscopy, FIA—fluorescent immunoassay, LOD—limit of detection, AUC—area under the curve, LLOQ—Linear Limit of Quantification. R^2^—coefficient of determination.

**Table 5:** SERS vs. fluorescence IA: median and geometric mean LODs for the most common analytes.

Analyte	SERS		Fluorescence		Year, Author
	Median LOD, M (Number of Works)	Geometric Mean LOD, M	Median LOD, M	Geometric Mean LOD, M	
Aflatoxin B1 (AFB1)	1.6 × 10^−10^ (2)	1.6 × 10^−11^ (2)	3.3 × 10^−10^ (2)	6.5 × 10^−11^ (2)	2023, Chen [98]; 2015, Ko [108]; 2020, Su [62]; 2019, Xie [69]
Ochratoxin A (OTA)	3.5 × 10^−12^ (2)	2.4 × 10^−12^ (2)	3.6 × 10^−13^ (2)	3.5 × 10^−13^ (2)	2023, Chen [98]; 2022, Jiao [106]; 2022, Chen [39]; 2020, Wang [56]
Alpha-fetoprotein (AFP)	6.0 × 10^−16^ (2)	7.3 × 10^−17^ (2)	3.2 × 10^−12^ (5)	2.0 × 10^−12^ (5)	2019, Yang [92]; 2017, Yang [109]; 2021, Sun [36]; 2017, Wang [38]; 2018, Chen [40]; 2019, Zhao [47]; 2021, Tian [74]
Cardiac troponin I (cTnI)	1.3 × 10^−13^ (3)	1.6 × 10^−13^ (3)	2.3 × 10^−11^ (2)	1.3 × 10^−11^ (2)	2021, Wang [85]; 2022, Gao [96]; 2019, Fu [101]; 2020, Liu [51]; 2020, Lee [67]
Median Value, M	1.82 × 10^−12^ (9)	1.28 × 10^−12^ (9)	1.31 × 10^−11^ (11)	7.50 × 10^−12^ (11)	

Abbreviations: SERS—surface-enhanced Raman spectroscopy, LOD—limit of detection.

**Table 6:** Comparison of relative standard deviations (RSDs) of studies with SERS-based and fluorescence-based immunoassay detection.

Method (Number of Works Involved in Calculations)	Average RSD, %	Median LOD, M
SERS-based IA (13)	6.2 ± 1.9	1.5 × 10^−12^
FIA (5)	5.4 ± 2.4	2.2 × 10^−11^

Abbreviations: RSD—relative standard deviation, SERS—surface-enhanced Raman spectroscopy, FIA—fluorescent immunoassay, IA—immunoassay, LOD—limit of detection.

**Table 7:** The literature review of challenges met in the application of FIA, and the proposed solutions.

Challenges	Solutions
The challenges and toxicity of the synthesis of QD probes for fluorescent immunoassays with the use of heavy metals such as cadmium and lead [41].	The use of carbon dots (CDs) with low costs, simplicity of preparation, good photostability, and lower toxicity [41].
Comparatively low sensitivity of the fluorescence immunoassays [52].	Use of alternative signal markers, such as fluorescent microspheres, carbon-based nanoparticles (NPs), and SERS nanomaterials [52].
Autofluorescence of the quantum dots from different substances [52].	Photobleaching of the quantum dots to reduce autofluorescence [146].

**Table 8:** A literature review of the challenges met in the application of SERS IA and proposed solutions.

Challenges	Solutions
Significant costs of gold film-covered substrates. Relatively high non-specific protein binding onto gold film and possible contamination of gold film with S-containing compounds	Use the Al and Si cost-effective substrates [32,137].
Relatively long assay time for stationary assays and sometimes relatively slow Raman measurements/readout	Use a wide-field Raman spectrometer; perform the analysis using the rotating substrate, microdroplet channels, strips and other alternative substrates, which significantly decrease the assay time [91,95,153,154].
Detection environment, including solution, temperature, and pH, may introduce an interfering species [154].	Use isolated systems like microdroplet channels and control the detection environment [91].
